# Deprescribing in Palliative Cancer Care

**DOI:** 10.3390/life12050613

**Published:** 2022-04-20

**Authors:** Christel Hedman, Gabriella Frisk, Linda Björkhem-Bergman

**Affiliations:** 1 Department of Molecular Medicine and Surgery, Karolinska Institutet, Anna Steckséns Gata 53, SE-171 76 Stockholm, Sweden; 2 R & D Department, Stockholms Sjukhem Foundation, Mariebergsgatan 22, SE-112 19 Stockholm, Sweden; 3 Department of Clinical Sciences Lund, Faculty of Medicine, Lund University, Scheelevägen 2, Medicon Village, SE-223 81 Lund, Sweden; 4 Department of Neurobiology, Care Sciences and Society (NVS), Division of Clinical Geriatrics, Karolinska Institutet, Blickagången 16, SE-141 83 Huddinge, Sweden; gabriella.frisk@ki.se (G.F.); linda.bjorkhem-bergman@ki.se (L.B.-B.); 5 ASIH Stockholm Södra, SLSO, Advanced Home Care and Palliative Care, Lasarettsvägen 4, SE-131 45 Nacka, Sweden; 6 Palliative Medicine, Theme Cancer, Karolinska University Hospital, SE-141 86 Huddinge, Sweden

**Keywords:** deprescribing, cancer care, palliative care, end-of-life, quality of life

## Abstract

The aim of palliative care is to maintain as high a quality of life (QoL) as possible despite a life-threatening illness. Thus, the prescribed medications need to be evaluated and the benefit of each treatment must be weighed against potential side effects. Medications that contribute to symptom relief and maintained QoL should be prioritized. However, studies have shown that treatment with preventive drugs that may not benefit the patient in end-of-life is generally deprescribed very late in the disease trajectory of cancer patients. Yet, knowing how and when to deprescribe drugs can be difficult. In addition, some drugs, such as beta-blockers, proton pump inhibitors, anti-depressants and cortisone need to be scaled down slowly to avoid troublesome withdrawal symptoms. In contrast, other medicines, such as statins, antihypertensives and vitamins, can be discontinued directly. The aim of this review is to give some advice according to when and how to deprescribe medications in palliative cancer care according to current evidence and clinical praxis. The review includes antihypertensive drugs, statins, anti-coagulants, aspirin, anti-diabetics, proton pump inhibitors, histamin-2-blockers, bisphosphonates denosumab, urologicals, anti-depressants, cortisone, thyroxin and vitamins.

## 1. Introduction

The aim of palliative care is to maintain as high quality of life (QoL) as possible. Thus, when patients enter a palliative phase, an evaluation of the on-going medication is recommended, and the benefit of each medication must be balanced against possible harm. The term used for the systematic process of tapering and discontinuing medications when existing or potential harms outweigh existing or potential benefits is “deprescribing” [1]. However, knowing how and when to deprescribe drugs can be difficult [2]. Preventive medications, such as statins or antihypertensives, could often be discontinued. The reason for wanting to lower cholesterol and blood pressure is to reduce the risk of cardiovascular events in a longer perspective. In patients with only a few months left in life, these factors matter less and usually do not affect the risk of cardiovascular events in the short term. However, the medications can cause troublesome side effects, especially when the patient deteriorates and the ability to eliminate drugs is impaired. Notably, previous studies have shown that medication with preventive drugs is generally terminated very late in cancer patients [3,4,5].

Taking a lot of medications in the end of life has been associated with high symptom burden and lower QoL according to previous reports [6]. A further aspect is that patients in end-of-life care often need medications against various symptoms [7] and thus deprescribing of unnecessary medication is important in order to avoid polypharmacy. In addition, the risk of drug interactions increases the more different drugs the patient takes. It is therefore important to prioritize the medicines that provide symptom relief here and now, such as pain relief, anxiolytics and antiemetics.

In some patients, deprescribing can be perceived as threatening and that doctors have “given up” about the patient [8]. It is therefore important to explain to both the patient and relatives that the rationale for the medication have changed that they are now doing more harm than good. At the same time, it is common for many patients to find it relieving to avoid many tablets [8]. To make the deprescribing process as safe and optimal as possible, without affecting the QoL of the patient negatively, it should preferably be conducted in agreement with the patient and/or relatives.

Appetite and nutrient intake are decreased at the end of life, and it can be difficult to swallow many tablets, especially large tablets. It is therefore good to consider not only the number of tablets but also the size of the tablets.

Some medicines should be de-escalated by slowly tapering the doses to avoid rebound effects, such as beta-blockers and proton pump inhibitors. A rebound effect may give harmful symptoms that can last for days to weeks. Some medicines need to be scaled down to avoid withdrawal symptoms, such as the discontinuation of antidepressants and cortisone. Other medicines can be discontinued immediately, such as statins. A summary of how to deprescribe different drugs are presented in Table 1.

To facilitate the deprescription of medication, several guidelines have been developed. The Screening Tool of Older Persons’ Potentially Inappropriate Prescriptions, the “STOPP Frail criteria”, are guidelines designed for frail, elderly patients with a short life time expectancy [9]. The general recommendations in the STOPP Frail-tool are to describe drugs that (i) the patient persistently fails to take or tolerate and (ii) drugs without a clear clinical indication. In addition, most drugs for primary prevention should be considered for deprescription if the patient has a short life-time expectancy.

The OncPal guidelines is another tool developed for helping physicians to limit numbers of inappropriate drugs in oncological patients in palliative care [10]. The tool includes drugs with limited or no beneficial effects in late-stage cancer disease but only lists those drugs where there is solid evidence for deprescription. Thus, medications not studied in this context are lacking.

The aim of this review is to give some advice about when and how to deprescribe medications in palliative cancer care according to current evidence and praxis.

## 2. Antihypertensives

The blood pressure usually decreases in cancer patients as the disease progresses and the need for blood pressure lowering medication is usually decreased in the late palliative phase [3]. In elderly patients, at the end of life, it has even been shown that slightly higher blood pressure is associated with longer survival time [11,12].

The vast majority of blood pressure medications can be discontinued straight off. Exceptions are beta blockers, described below. If the indication of the medication is heart failure it is better to de-escalate to avoid increased symptoms such as dyspnea or edema. Loop diuretics as intravenous administration may be helpful for symptomatic patients with dyspnea due to end-stage heart failure instead of tablets in the end-of-life setting [13]. Loop-diuretics for parenteral administration (iv or sc) is also an important drug for symptom management in the dying patient in case of hyperhydration or respiratory distress due to pulmonary oedema.

### 2.1. Beta-Blocking Agents

Beta blockers should, if possible, be carefully scaled out to counteract rebound effects since there is usually an increased sensitivity to catecholamines after long-term treatments. A too rapid termination can cause tachycardias, anxiety, tremor and sweating. It is recommended to decrease the dose by 50% for 1 week and then an additional 50% reduction for another week until the lowest possible dose is reached for that particular drug preparation. After treatment with the lowest dose for one week, the drug can be discontinued [14].

### 2.2. ACE-Inhibitor

When treating with ACE inhibitors for preventing cardio-vascular disease, such as hypertension, there is no indication for further prescription when you have a short life expectancy due to another uncurable disease. The treatment can generally be stopped immediately without any risks shortly thereafter. However, you can have a slow rise in blood pressure over a few weeks and it could therefore be important in some cases to monitor the blood pressure 2 to 4 weeks after the treatment has ended [15].

For other indications, such as heart failure, ACE inhibitors should be scaled down with a careful supervision of the clinical picture. It is important to stop the scaling out if you see a change in symptoms in the patient that motivates further treatment. Patients with severe heart failure should continue with ACE inhibitors as long as possible with the lowest possible dose for optimal symptom relief, if the patient can take tablets [16].

## 3. Statins

There are no clear guidelines to support when statins should be terminated in end-of-life patients [17]. However, studies from palliative care show that statins are generally safe to discontinue in cancer patients with an expected survival time of 1 year or less [18,19]. In addition, geriatric patients with life-limiting diseases seem to be more susceptible to statin induced myalgia and pain [20].

Kutner and co-workers performed a randomized, unblinded clinical trial on statin discontinuation in 381 patients admitted to palliative care and with an expected survival time between 1 year to 1 month. This study showed that there was no statistically significant difference in time to death between patients who continued or those who terminated treatment, and there was no difference in cardiovascular events between the groups [19]. Notably, the patients randomized to the discontinuation of statins assessed their QoL higher than those continuing [19]. This is in line with two observational studies in palliative cancer care, where early discontinuation of statins was not associated with increased risk of cardiovascular events [18,21] and maintained QoL in these patients [18]. Both these studies also showed a sex-difference in statin termination where women in palliative cancer care generally had their statins deprescribed earlier in their disease trajectory than men [18,21].

In contrast, other studies have shown that in patients with unstable cardiovascular disease and after a cardiovascular event, stroke or myocardial infarction, statin treatment should be maintained if possible [22,23].

It should be noted, however, that several studies have indicated that statin use may be beneficial in cancer disease [24,25,26,27]. However, today, there are no general recommendations to continue statin treatment for cancer-preventive effects. In addition, possible beneficial effects of statin in cancer disease is probably of limited importance in the late palliative phase.

Usually, statins can be terminated straight off and do not need to be scaled down, since the risk of rebound effects is small.

## 4. Anticoagulation

Anticoagulants are prescribed for primary prevention, treatment and secondary prevention of stroke and venous thromboembolism (VTE). Several different anticoagulants, warfarin, low-molecular-weight heparins (LMWH) and direct oral anticoagulants (DOAC) are available [28]. During end-of-life care, patients have increased risk of thromboembolic diseases, due to, e.g., immobilization and metastatic cancer disease. One study found that approximately 10% of cancer patients hospitalized in palliative care units had deep vein thrombosis (DVT) [29].

The use of anticoagulants is challenging, as symptoms from thrombosis, such as stroke, swelling of a leg or dyspnea, can affect QoL, and at the same time, the risk of bleeding is increased during end-of-life. Thus, the decision of deprescription of anticoagulation therapy should be based on the reason of prescription, i.e., prophylaxis or treatment. In addition, the form of administration can affect the decision, although patients seem to accept subcutaneous injections as a treatment option even late in the disease trajectory. Tapering of the doses could be important in patients losing weight, as LMWH doses are dependent on weight. DOACs have several drug–drug interactions and are not recommended to patients with renal and hepatic failure, thus limiting their use in these patients. The use of warfarin requires regular laboratory testing and has multiple drug–drug interactions, making it less optimal as well.

Based on guidelines from the National Institute for Health and Care Excellence, VTE prophylaxis with LMWH could be considered for patients during palliative care. However, the decision to treat must be balanced with the risk of bleeding and estimated life expectancy. Preferably, the decision should be taken together with the patient and their family. VTE prophylaxis is not recommended in the last days of life [30].

Prophylaxis of stroke in patients with atrial fibrillation and mechanical valve disease are not recommended in end-of-life care due to the increased risk of bleeding and relatively low risk of stroke. However, no studies regarding deprescription are available [31,32].

On the other hand, those with a recent thromboembolic disease, anticoagulation, preferably with LMWH, should be continued longer and could be deprescribed during the last days to weeks. Only those with a recent, and major thromboembolic event, such as a lung embolism, should have LMWH until the last days if life [33].

## 5. Aspirin

Aspirin (ASA) has several indications such as primary and secondary prevention in coronary heart disease, arterial fibrillation, peripheral arterial disease and for secondary prevention after stroke. Aspirin is generally more well-tolerated with less adverse effects than other anti-coagulants. In addition, it is a small tablet that is easy to swallow. However, in stable heart disease, aspirin can usually be deprescribed the last month in life and for primary prevention even earlier. Aspirin is an irreversible inhibitor of cyclooxygenase in platelets and prevent them from aggregation. Since the effect on platelets is permanent, the effect remains throughout the life cycle of the platelet, which is 7 to 10 days. Thus, the treatment can be terminated straight off and still have an effect for at least one week.

The balance between beneficial effects and possible harm, i.e., bleedings, has to be assessed in each individual. In a recent study, it was shown that there was an over-prescription with co-medication of both ASA and DOACs [34]. Interestingly, the combined therapy of the two anti-coagulants was associated with increased rate of severe bleedings and hospital stays and still no difference in thrombosis rate was observed compared to those with only one anti-coagulant. Although the conclusions in this study [34] have been debated the important notion is that a clear indication and rationale for the different treatments that patients receive are crucial [35].

## 6. Anti-Diabetics

Keeping a strict control of B-glucose is not as necessary in end-of-life patients since the long-term effect of hyperglycemia is no longer a problem and slightly elevated B-glucose levels usually do not cause distressing symptoms. In contrast, hypoglycemia should always be avoided since this causes both worrisome symptoms and may even shorten life. Thus, the blood glucose should not be pushed too low. However, no evidence-based guidelines are available for the treatment of diabetes mellitus in end-of-life care.

Oral antidiabetics can often be discontinued when the patient is in a late palliative phase to avoid the risk of side effects [10], and switching to only insulin is a good option when anti-diabetics are still needed. Often, the need for insulin at the end of life also decreases due to decreasing weight and as the patient eats less and eventually receives no nutrition at all. In case of symptomatic hyperglycemia, insulin can be given. In patients with type 1 diabetes insulin might be needed late in the disease trajectory.

## 7. Proton Pump Inhibitors

Proton pump inhibitors (PPI) (e.g., omeprazole, lansoprazole) should be scaled down if the treatment has lasted more than a month in order to avoid rebound effects [36,37]. Long-term use of PPIs results in an increased production of gastric acid when treatment is stopped [36,37]. Reducing the dose to half of the original dose for two weeks before cessation reduces the risk of withdrawal symptoms. At the dose 20 mg omeprazole/day, 10 mg/day or 20 mg can be given every other day.

Oncological patients are often treated with PPIs for primary preventive purposes to protect the gastric mucosa against ulcers during long-term treatment with cortisone and/or NSAIDs. In most cases, omeprazole 20 mg is quite enough to achieve good protection. Higher doses should be avoided to counteract potential withdrawal symptoms when discontinuing treatment.

## 8. Histamin-2-Blockers

For histamin-2-blockers there is also a risk of rebound effects when the treatment is terminated due to hypersecretion of gastric acid [38]. To avoid this, it is recommended to reduce the dose to half of the original dose for two weeks and then terminate the medication. If the treatment time has been shorter than 1 month it can usually be deprescribed straight off.

Histamin-2 blockers should generally be avoided in elderly and fragile patients, such as end-of-life patients, due to increased risk for cognitive impairment. If gastric acid inhibition is needed in these patients, PPI is generally the first-hand choice.

## 9. Bisphosphonates and Denosumab

Osteoporosis in geriatric patients should be treated since fractures due to osteoporosis are painful and are associated with reduced quality of life as well as shorter life expectancy. Bone resorption inhibitors, such as bisphosphonates and denosumab have effect already after a short treatment period and can therefore be given to patients with a relatively short expected survival. However, patients with an expected survival for less than one year, or who are bound to bed do not need to take bisphosphonates if the indication is osteoporosis. Prophylactic treatment for osteoporosis treatment should generally be given to patients receiving cortisone therapy (equivalent to >5 mg prednisolone/day for at least 3 months), unless the expected survival is less than one year. Treatment with bisphosphonates, calcium and vitamin D can be discontinued immediately [39,40].

Bisphosphonates are also used for symptomatic treatment for bone metastasis and may reduce pain and prevent pathological fractures. For this indication, bisphosphonates can be continued until later in the disease trajectory but are usually discounted if the expected survival time is less than 1 month.

Bisphosphonates can also be used for treatment of hypercalcemia in palliative care patients and can with that indication be given also in a late phase of the disease trajectory to relieve symptoms.

Denosumab is a more recently developed drug that can be used for the same indications as bisphosphonate and is given as sc injections. It is an alternative in patients where bisphosphonate cannot be prescribed due to contraindications, e.g., impaired renal function (eGRF < 30 mL/min). However, when denosumab is discontinued, there is a risk for a rebound effect that can last up to two years, leading to bone loss and increased risk of fractures. For cancer patients with bone metastases, there is also a risk for increased pain after discontinuation. To prevent this, it is recommended to give a single dose of zoledronic acid, if it is tolerated, six months after the last dose of denosumab [41,42] or to continue denosumab to a late stage of the disease.

## 10. Urologicals

Lower urinary tract symptoms (LUTS) refer to a group of urinary symptoms categorized into storage or obstructive symptoms. Medications used for these symptoms include anticholinergics (tolterodine, solifenacin, oxybutynin, darifenacin and fesoterodine), serotonin-noradrenaline reuptake inhibitor (duloxetine), antidiuretic hormone (desmopressin), alpha-1 blockers (doxazosin, urapidil, terazosin) and 5-alpha-reductase inhibitors.

In end of life, anticholinergics should be avoided, especially in the elderly, due to the risk of cognitive impairment, urine retention, dryness of the mouth and obstipation [43]. Duloxetine has a questionable effect on LUTS and have increased risk of adverse effect in patients with kidney failure [43,44]. Desmopressin has only weak effects on LUTS and poses a risk of hyponatremia. Both these medications should thus be considered for deprescription in late-stage palliative care patients [43].

Peripheral alpha-1 blockers could be deprescribed due to limited clinical effects and an increased risk of orthostatic hypotension with subsequent risks of fall injuries. In addition, they cause increased urine incontinence in women and should thus be avoided in end of life. The 5-alpha-reductase inhibitors are often prescribed with a questionable indication and should be considered for deprescription [43,45]. In patients with urinary catheters, deprescription of urological medications should be considered to be deprescribed due to questionable effects [46].

## 11. Anti-Depressants

Depressions and depressive symptoms are common in patients facing a life-threatening illness. An untreated depression will contribute to symptom burden in palliative care patients and make symptom management more difficult. Thus, it is important to identify and treat depressive disorders in palliative care patients in order to maintain as high QoL as possible. In line with this, previous studies have shown that anti-depression drugs have beneficial effects also in late-stage palliative patients [47]. However, most antidepressants do not exist for parenteral administration, so if and when the patient can no longer swallow, it has to be discontinued.

When antidepressants are discontinued, especially serotonin uptake inhibitors (SSRIs), they need to be carefully scaled out to avoid withdrawal symptoms. Withdrawal symptoms of SSRIs include dizziness, anxiety, nausea, blurred vision, sleep problems, headaches and paresthesia. These symptoms can be avoided if the dose is carefully reduced before termination. Since SSRIs do not exist for parenteral administration, it is optimal if SSRIs can be reduced to the lowest effective dose in end-of-life. It may also be needed to be replaced with anxiolytics, preferable benzodiazepines, to counteract anxiety and withdrawal symptoms. The benzodiazepine midazolam can also be administered sc or iv and can thus be used to the very end of life.

## 12. Corticosteroids

Corticosteroids is a drug widely used in palliative care due to broad and useful effects in symptom management, including anti-inflammatory properties and positive effects on, e.g., fatigue, pain, nausea and appetite. However, the effect deteriorates during long-term use, and it is optimal to use for only shorter time-periods, e.g., 2 weeks. Interestingly, although the extensive use of corticosteroids, general guidelines for the use of this drug in palliative care is lacking [48]. Indeed, in two recent meta-analyses and reviews, it is shown that the evidence base for most indications of corticosteroids in palliative care were limited or non-existing [48,49].

In long-term use of cortisone, it should, if possible, be scaled down to the lowest effective dose and carefully scaled down at the end of life to avoid withdrawal symptoms. Treatment that has lasted for two weeks or less can usually be discontinued immediately without de-escalation. However, many cancer patients use cortisone for a longer period, and thus there is a risk of adrenal insufficiency if cortisone is deprescribed. The symptoms of adrenal insufficiency can mimic those during severe illness, such as fatigue, weight loss and pain, and cortisone treatment should continue in these patients [50].

Cortisone treatment can be maintained until the end of life if there is a strong symptom-relieving indication, such as counteracting brain edema in primary brain tumors or brain metastases, pain relief where other pain relief has not had an effect and as nausea treatment where other antiemetics have not had an effect (e.g., in malignant ileus) [7,51]. If the patient is unable to swallow or has no uptake from the intestine (as in ileus), cortisone can be given as injections iv or sc once daily. Most often, it can be discontinued in the last 24 h without withdrawal symptoms because the effect in the body remains for more than 48 h after the administered dose.

When corticosteroids are prescribed, blood glucose monitoring might be needed to avoid symptoms from high blood glucose.

## 13. Levothyroxine

Lifelong replacement therapy with levothyroxine is indicated in persistent thyroid hormone deficiency, related to, e.g., thyroiditis, surgery or radiotherapy. Levothyroxine is normally administered by tablets but is also available as oral liquid and intravenous formulation in many countries. Appropriate dosing is driven by laboratory monitoring of thyroid stimulating hormone (TSH). As the dose is partly weight dependent, loss of weight might affect the dose needed and monitoring of TSH is important in patients with severe illnesses. The half-life of levothyroxine is approximately 7 days. Thus, a short interruption in administration of levothyroxine does not give symptoms of hypothyroidism [52,53], but more than a couple of weeks’ interruption will give symptoms. Notably, these symptoms are similar to those during end of life, such as fatigue, weakness, cognitive dysfunction, constipation and edema. Symptoms from hypothyroidism have been described in patients during hospice care, and these symptoms could thus potentially impair QoL in end-of-life care [54].

Levothyroxine is one of the most common medications in seriously ill patients, and deprescription is thus an important issue [55]. It is recommended to continue with levothyroxine as long as possible, especially in those with high levothyroxine doses and in those with no thyroid hormone productions. In rare cases, when a patient is unable to swallow for more than weeks or there is no uptake from the intestines, an intravenous formulation could be an alternative.

## 14. Vitamins and Minerals

Most often, vitamins can be terminated in the last months of life. If the patient has been treated with vitamins for a long time, there are usually depots that last for several months. In some oncology treatments, such as pemetrexed, B12 and folacin are needed during treatment to reduce side effects and should thus not be terminated.

A recent randomized, placebo-controlled and double-blind study has shown that vitamin D treatment can reduce pain and fatigue in palliative cancer patients with vitamin D deficiency, defined as 25-hydroxyvitamin D < 50 nmol/L [56]. However, the effect was not very large, but the high dose of 4000 IU/day was well tolerated in end-of-life patients with no increased risk of hypercalcemia. The effect of the treatment was significant first after 12 weeks and thus, in patients with a shorter survival time, the treatment had no benefit [56]. Interestingly, the beneficial effect of vitamin D on fatigue seems to be more pronounced in men than in women [57].

Supplementation of minerals can generally be described during the last months in life. Calcium especially might be considered for early deprescription since there is an increased risk for hypercalcemia in late-stage cancer patients.

In Table 2, a summary for different medications and their potential deprescription strategies is provided. However, the decision must always be based on the individual situation of the patient.

## 15. Conclusions

Thoughtful deprescribing of medications without symptom relieving effects during end-of-life is important to maintain as good a QoL as possible. Several unnecessary medications can be withdrawn, whereas others, such as corticosteroids and PPIs, should be slowly tapered. In palliative cancer patients, some important issues should be remembered, such as the need of corticosteroids until the end of life if they relieve symptoms and the need of anticoagulation in patients with recent thrombosis. Thus, deprescription requires information of medications and thorough knowledge of the patients. Finally, deprescription requires a careful discussion with patients and relatives.

## Figures and Tables

**Table 1:** How to deprescribe different drugs in end-of-life patients.

Drug	De-Escalation Recommended	Discontinue Straight Off
Statins		X
Anihypertensives		X
Vitamins		X
Urologicals		X
Bisphosphonates		X
Corticosteroids < 2 weeks		X
Corticosteroids > 2 weeks	X	
Anti-depressants	X	
Proton pump inhibitors	X	
Beta blockers	X	

**Table 2:** Suggested time when deprescribing of drugs can be started to be considered (X) in cancer patients admitted to palliative care.

	Expected Remaining Survival Time			
Drug	<1 Year	<3 Months	<1 Month	Days
Statins	X	X	X	X
Anihypertensives		X	X	X
Urologicals		X	X	X
Vitamins		X	X	X
Anti-coagulants		Individual assessment		
Anti-diabetics, p.o.			(X)	X
Anti-depressants				X
Thyroxin				X
Corticosteroids				X
Bisphosphonates		Depends on indication		
